# Structural development and brain asymmetry in the fronto-limbic regions in preschool-aged children

**DOI:** 10.3389/fped.2024.1362409

**Published:** 2024-10-01

**Authors:** Gang Yi Lee, Young-Ah Youn, Yong Hun Jang, Hyuna Kim, Joo Young Lee, Young Jun Lee, Minyoung Jung, Hyun Ju Lee

**Affiliations:** ^1^ Department of Translational Medicine, Hanyang University Graduate School of Biomedical Science and Engineering, Seoul, Republic of Korea; ^2^ Department of Pediatrics, College of Medicine, Seoul St. Mary's Hospital, The Catholic University of Korea, Seoul, Republic of Korea; ^3^ Department of Radiology, Hanyang University Hospital, Hanyang University College of Medicine, Seoul, Republic of Korea; ^4^ Cognitive Science Research Group, Korea Brain Research Institute, Daegu, Republic of Korea; ^5^ Department of Pediatrics, Hanyang University Hospital, Hanyang University College of Medicine, Seoul, Republic of Korea; ^6^ Division of Neonatology and Development Medicine, Hanyang University Hospital, Seoul, Republic of Korea; ^7^ Hanyang Institute of Bioscience and Biotechnology, Hanyang University, Seoul, Republic of Korea

**Keywords:** brain development, fronto-limbic, preschool-aged children, volume, thickness, asymmetry, structural MRI

## Abstract

Early-life experiences play a crucial role in the development of the fronto-limbic regions, influencing both macro- and microstructural changes in the brain. These alterations profoundly impact cognitive, social-emotional functions. Recently, early limbic structural alterations have been associated with numerous neurological and psychiatric morbidities. Although identifying normative developmental trajectories is essential for determining brain alterations, only a few studies have focused on examining the normative trajectories in the fronto-limbic regions during preschool-aged children. The aim of this study was to investigate the structural-developmental trajectory of the fronto-limbic regions using the cortical thickness, volume, and subcortical volume in 57 healthy and typical preschool-aged children between 1 and 5 years and examined the early lateralization patterns during the development of the fronto-limbic regions. Regarding brain lateralization, remarkable asymmetry was detected in the volume of thalamus and the cortical regions excluding the lateral orbitofrontal cortex in the fronto-limbic regions. This study of preschool-aged children may fill the knowledge gaps regarding the developmental patterns and hemispheric asymmetries of the fronto-limbic regions between newborns and adolescents.

## 1 Introduction

Brain development is a complex process that includes micro- and macrostructural changes (1, 2), accompanied by regional structural brain changes. Structural cortical development begins in the fetal and changes significantly throughout life, overlapping nonlinear trajectories (3, 4). Cortex maturation involves its integration into the higher-order brain areas such as the frontal cortices following the development of lower-order somatosensory and visual cortices. This leads to an intricate and asynchronous developmental pattern, with the sequence influenced by phylogenetic factors and anatomical location (5). These brain morphological and microstructural changes can be indirectly quantified through MRI (6–8).

Cortical volume generally follows a logarithmic growth pattern in most regions over the preschool-aged range, reflecting the maturation and organization of neural connections and structures that underpin cognitive development (9). However, frontal and cingulate brain regions followed a quadratic trajectory with peak volume values obtained around 5–6 years of age (10). The phenomenon of cortical thinning is closely tied to the concept of neural connections such as synaptic pruning and intracortical myelination (2, 11). The development trajectories of cortical thickness differ across the brain in their functions and locations. The pattern of cortical thickness development in chronological development shows the largest peak at 1–2 years of age (12), followed by a logarithmic decreasing pattern in most regions until 6 years of age (13). However, that of several regions in the frontal and posterior regions exhibited increasing pattern, following a quadratic trajectory during childhood (10, 14).

The age of 1–5 years is considered a critical period for the emergence and development of various cognitive functions, with dynamic brain structural changes (15). The fronto-limbic regions were established and evolved into a crucial neural network as a critical process in early neurodevelopment (16). The period from 1 to 5 years old holds significance as it coincides with the emergence and initial refinement of the fronto-limbic circuit (17, 18). Moreover, it is the time when numerous developmental, behavioral, and intellectual disorders are thought to initially manifest (19, 20). Social cognitive functions are shaped by the neural network structure of the fronto-limbic regions, which is organized through complex interconnections between the frontal and subcortical regions (21). Although cognitive development is better understood through systemic changes in functional connectivity among various core regions (22), it is important to note that the fronto-limbic regions significantly contribute to diverse cognitive processes such as executive functions, decision-making, attention, and emotional regulation. Of note, many of the socio-emotional disorders are believed to arise during early neurodevelopment (19, 23) and are likely to be associated with or caused by abnormal cortical development of the fronto-limbic regions (24).

The early development of brain lateralization exhibits a multivariate nature, influenced by various exogenous factors, and raises questions regarding the generalizability of early brain lateralization patterns (25–27). The lateralization of the fronto-limbic regions appears to be organized for specialization in processing social information and emotional functioning in both children (28) and adults (29). Our previous study demonstrated the altered asymmetry of neonatal brain in the fronto-limbic regions connection is associated with social–emotional scores at 18 months of age, using diffusion tensor imaging tractography and structural network analysis. Alterations in lateralization within the frontal cortex, specifically related to reduced asymmetry in cortical thickness, could play a significant role in the early onset of autism spectrum disorder (ASD) in childhood (30). Greater asymmetry of the anterior cingulate correlated with a higher score inattention subscale of Barratt Impulsiveness Scale in adult patients with personality disorder than healthy controls (23). Recent studies show that an abnormal asymmetry in cortical thickness and volume may be linked to various neuropsychiatric conditions, such as attention deficit hyperactivity disorder (11, 31), schizophrenia (32, 33), and autism spectrum disorder (11). Understanding the developmental trajectory of cortical asymmetry and its regional variations may contribute to identifying deviations that may be associated with neurodevelopmental disorders or cognitive impairments.

Although brain lateralization of cortical volume (19, 34) and thickness (35, 36) has been extensively studied over the past few years, the intricate patterns of lateralization in children across the fronto-limbic regions remain a complex issue that has not been fully elucidated. The direction and magnitude of lateralization in the fronto-limbic regions during critical stages of childhood, as an evolutionary adaptation, remain unclear. Furthermore, owing to the challenges associated with acquiring and processing infant brain MRI data, our understanding of the evolving patterns of cortical volume and thickness and the age-related change in hemispheric lateralization is currently limited.

We investigated the structural-developmental trajectory and early lateralization patterns of the fronto-limbic regions by measuring the cortical thickness, volume, and subcortical volume in typically developing preschool-aged children aged between 1 and 5 years.

## 2 Materials and methods

### 2.1 Clinical characteristics

Eighty healthy and typically developing children aged 1–5 years were recruited from Hanyang University Hospital in Seoul, Korea (Supplementary Figure S1). Children who underwent a detailed neurological examination and developmental assessment conducted by a pediatrician at the Hanyang Inclusive Clinic for Developmental Disorders were recruited for this study between 2018 and 2021. The Institutional Review Board of Hanyang University Hospital approved the protocol and scanning procedures of this study, and informed consent was obtained from the parents of all children participating in the study. Developmental screening was performed during a routine health checkup using the Korean-Developmental Screening Test (K-DST) for children at the Hanyang University Medical Center Pediatrics Department. Children with developmental scores within 2 standard deviations of the mean on all subscales of the K-DST were included. We excluded 3 preschool-aged children with signs of developmental delay and known risk factors for abnormal development, such as complications during pregnancy, a family history of psychiatric or neurological disorders, pervasive developmental disorders, congenital malformations, chromosomal anomalies, and neurological events or disorders (e.g., head trauma or epilepsy). Also, 20 children were excluded from image quality check analysis due to motion artifacts and poor image quality. Finally, we used data from 57 children aged 14–71 months (Table 1).

**Table 1:** Participant demographic information.

	Age 1 (*n* = 7)	Age 2 (*n* = 15)	Age 3 (*n* = 14)	Age 4 (*n* = 11)	Age 5 (*n* = 10)	*p-value*	*LSD*
Mean scan age, months	18.00 ± 3.16	30.53 ± 3.91	40.64 ± 3.73	54.09 ± 2.98	64.80 ± 2.86	**<0**.**001**	1 < 2 < 3 < 4 < 5
Male (%)	4 (57.1)	11 (73.3)	12 (85.7)	6 (54.6)	8 (80.0)	0.398	
Birth weight (g)	3,387.14 ± 670.52	2,864.00 ± 690.55	2,754.29 ± 767.41	3,137.27 ± 253.70	3,197.00 ± 412.37	0.130	
Maternal age	30.29 ± 4.35	34.73 ± 3.03	34.36 ± 5.00	33.64 ± 3.85	32.00 ± 5.01	0.151	

Data are represented as the mean ± standard deviation, and significant group differences (*p* < 0.05) are highlighted in bold.

LSD, least significant difference.

### 2.2 MRI acquisitions

Individuals underwent a 3 T MRI scan (Philips, Achieva, 16-channel phase-array head coil, Best, Netherlands) without sedation. On T1-weighted images, the single-shot three-dimensional echo-planar images were acquired using the following parameters: slice thickness = 1 mm, voxel sizes = 0.9 mm^2^, field of view = 224 mm^2^, repetition time = 8.3 ms, echo time = 4.6 ms, inverse time = 1 ms, and flip angle = 8°. The slice orientation was axially parallel to the anterior-posterior commissure line.

### 2.3 Image preprocessing

Automated reconstruction and segmentation of T1-weighted images were conducted using FreeSurfer version 7.2.0. This pipeline enables motion correction, automated Talairach transformation, non-brain tissue removal, signal intensity normalization, automated topological defect correction, subcortical segmentation, and cortical parcellation. To reduce the influence of low-frequency signal intensity nonuniformity when generating the original surface, bias field estimation was performed on the T1-weighted images using the N4 bias field correction of Advanced Normalization Tools (37). The cerebral cortex was parcellated into 68 anatomical regions (34 bilateral regions) according to the Desikan-Killiany Atlas.

### 2.4 Image quality check

Due to the challenges associated with cortical surface parcellation in the developing brain, two independent researchers conducted automatic and manual quality assessments in five steps on all reconstructed imaging data (Supplementary Figure S2). The initial visual inspection categorized motion artifacts, ghosting, and ringing in the raw imaging data into three ratings: good, moderate, and bad (38–40) (Supplementary Figure S3). Of these, data in the bad ratings was excluded from further processing.

Given the rapid and considerable development of the brain during preschool-aged children, special consideration is essential to examine the contrast imaging and brain morphology in preschool-aged children (26). In this study, we identified the remaining images that underwent visual assessment using Quality Assurance (QA) tools and ENIGMA (Enhancing Neuro Imaging Genetics through Meta-Analysis) algorithms, generating quantitative and qualitative information regarding the image quality. The two automatic assessments are as follows: First, all subcortical segmentation of regional volumes was evaluated through the QA tools to identify the potential outliers at the individual level within the population. Second, we plotted the cortical surface segmentation to provide qualitative information by combining snapshots of inner and outer slices through ENIGMA algorithms.

Additionally, errors with brain segmentation may arise because of inaccuracies in children's white matter (WM) intensity during intensity normalization. To avoid this, we used control points manually to regulate the WM hypointensities for identified subjects, keeping it within the range of 80–110 (27). Finally, to maximize accuracy, two independent researchers conducted visual inspections of the reanalyzed images to select the final set of images for inclusion.

### 2.5 Image processing

Preschool-aged children with neurological disorders, history of neurological or psychiatric disorders, or negative results on brain MRI were excluded. Our study focuses on the most relevant regions of interest, specifically within the fronto-limbic neural circuitry. The 11 regions of interest were chosen based on previously identified areas associated with executive function, decision-making, attention, and emotion regulation (16, 41, 42). The region of interest (ROI)s used in this study were the 11 fronto-limbic regions: the subcortical regions of the thalamus, amygdala, and hippocampus; the rostral anterior cingulate cortex (rostral ACC), caudal anterior cingulate cortex (caudal ACC) and posterior cingulate cortex (PCC) through the medial and lateral orbitofrontal cortex (medial OFC and lateral OFC); and the superior temporal gyrus (STG), inferior parietal cortex (IPC), and fusiform. ROI approach was used to extract the fronto-limbic regions according to the Desikan-Killiany Atlas. As a significant correlation was observed between the regional volume measurements and the estimated total intracranial volume (eTIV), all the regional volumes were divided by eTIV, and multiplied by 1,000 to normalize the individual variations in skull size to reduce interindividual variation and minimize subtle distortions in brain imaging. The relative volume was determined as follows:

$$\mathrm{Relative}\;\mathrm{volume}=\frac{\mathrm{Region}\;\mathrm{of}\;\mathrm{interest}}{\;\mathrm{eTIV}}\times 1,000$$

For cortical thickness, we analyzed the widely used absolute value and obtained the relative cortical thickness for each area of the brain by dividing it by the mean cortical thickness within the same hemisphere.

$$\mathrm{Relative}\;\mathrm{thickness}=\frac{\mathrm{Region}\;\mathrm{of}\;\mathrm{interest}}{\mathrm{Mean}\;\mathrm{thickness}}$$

### 2.6 Statistical analysis

To evaluate cortical maturation with age between 1 and 5 years, we used a generalized additive model (GAM) adjusting for sex to detect nonlinear patterns between age and brain measures. The GAM is ideal for addressing the internal dependency structure of the data, including regional correlations in volume and thickness, as well as potential non-Gaussian distributions (43, 44). GAM effectively captures nonlinearities and variations, allowing for flexible and precise estimation of nonlinear effects. We have applied the GAM model to investigate the relationship between cortical measurements and age (in months) over a 5-year period. This method is particularly valuable for analyzing the rapid developmental changes in preschool-aged children, where complex nonlinear patterns arise due to both macrostructural and microstructural brain changes. The R package (45) was used to visualize the non-linear trajectory across all ages. Additionally, we evaluated the Bayesian information criterion to identify the most parsimonious model fit. For multiple comparisons, the significant *p*-value was adjusted using FDR-corrected across eight brain regions for cortical thickness and eleven brain regions for volume, respectively.

An asymmetry index (AI) was calculated to characterize lateralization during cortical maturation, determining the thickness and volume of 11 fronto-limbic brain regions for each individual. The following equation was used:

$$\mathrm{AI}=\frac{\left(\mathrm{Left}-\mathrm{Right}\right)}{\left(\mathrm{Left}+\mathrm{Right}\right)}$$

The AI values of the two measures were analyzed using a sliding window approach with age to identify the specific age intervals of significant asymmetric cortical development. By ordering the participants according to the number of months, the average AI at 12-month intervals was calculated. The Wilcoxon signed-rank test (46) was used to measure the level of left-right lateralization of the groups, with the null hypothesis centered on an asymmetric mean of zero.

## 3 Results

### 3.1 Brain tissue volumes

The sample (*n* = 57) in this study was included 41 male and 16 female. Supplementary Figure S4A shows the eTIV of the five age-groups. The eTIV exhibited an increasing pattern from 1 to 5 years. No significant year-by-year change was observed among 5 groups, aged 1 to 5 years (*p* = 0.180, *p* = 1.000, *p* = 0.213, and *p* = 0.756, respectively). However, significant difference was observed between the ages 1 and 4 years and between the ages 1 and 5 years (*p* = 0.023 and *p* = 0.049, respectively). The relative and absolute values (original volume × 1,000/eTIV) of the five groups in the total gray matter, subcortical gray matter, cerebral cortex, and cerebral WM. They are shown in Supplementary Figure S4B and Table S1, respectively.

### 3.2 Maturation trajectories of the fronto-limbic volume and thickness

To characterize maturation in the fronto-limbic thickness and volume from 1 to 5 years, GAM models were fit to the data. In relative cortical volume, we found that cortical volume followed diverse trajectories with gradual increases throughout the fronto-limbic regions (Supplementary Figures S5A,B). Additionally, growth curves in relative cortical thickness showed a progressive decreasing pattern compared to cortical volume across age (Supplementary Figure S6). Also, the subcortical volume was found to increase over the preschool-aged range (Supplementary Figure S5C).

**Figure 1:**
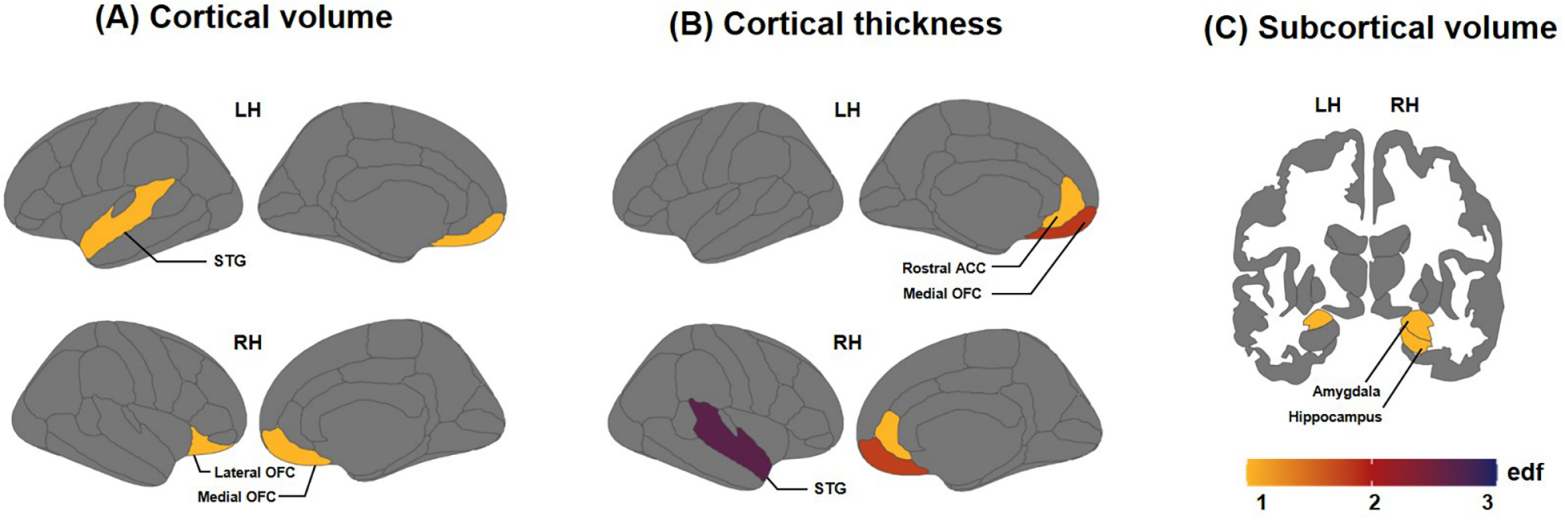
Effective degree of freedom (edf) values of brain regions showing significant correlations between brain measures and age (months). The edf reflects the degrees of curvature of smooths: **(A)** edf values for cortical volume, **(B)** edf values for cortical thickness, and **(C)** edf values for subcortical volume. Edf = 1 indicates a linear relationship; edf > 1 signifies more intricate associations between brain measures and age.

The results of GAM models revealed that the effective degree of freedom (edf) values, which indicate model complexity, were significantly correlated with age in some brain regions in cortical thickness, and subcortical volume (Figure 1). We observed that the cortical thickness revealed significantly decreasing pattern in the right rostral ACC (edf = 1.0, F = 6.906, *p* = 0.032); medial OFC in both the left (edf = 1.839, F = 11.88, *p* < 0.001) and right (edf = 1.792, F = 6.542, *p* = 0.016) hemispheres; and an increasing pattern in the right STG (edf = 2.651, F = 4.56, *p* = 0.032). Subcortical volume, which is significantly pronounced in preschool-age children in the right hippocampus (edf = 1.0, F = 11.65, *p* = 0.006), and amygdala in both the left (edf = 1.0, F = 10.74, *p* = 0.022) and right (edf = 1.0, F = 19.04, *p* = 0.001) hemispheres. The edf results and statistical values of each brain region are shown in Supplementary Tables S2 and S3.

### 3.3 Asymmetries of the fronto-limbic volume and thickness

**Figure 2:**
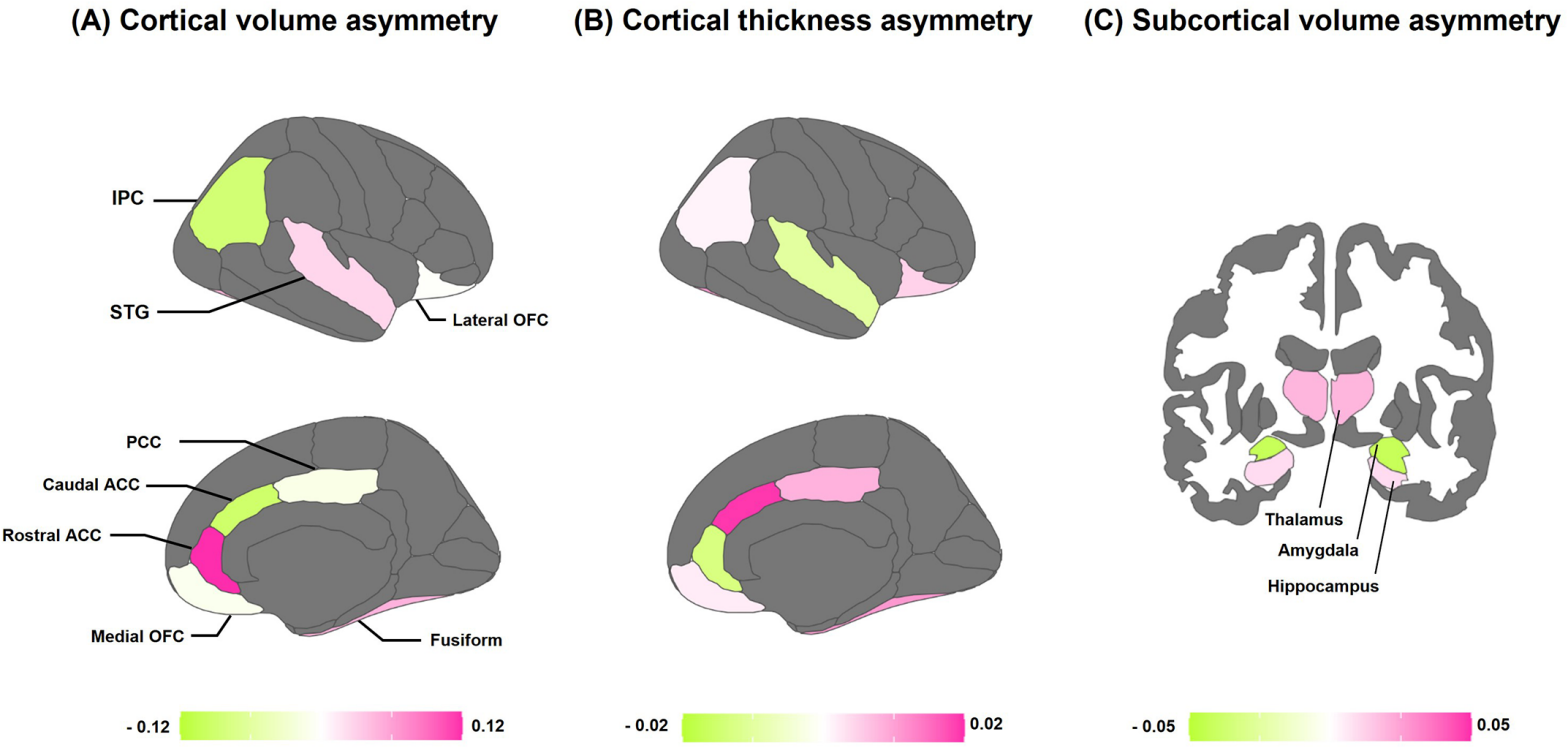
Average asymmetries of the fronto-limbic regions. **(A)** Population average regional asymmetries of cortical volume. **(B)** Population average regional asymmetries of cortical thickness. **(C)** Population average regional asymmetries of subcortical volume. Colors indicate the directions of average interhemispheric differences, with pink indicating left-ward asymmetry (i.e., a greater left-ward than right-ward measure), and green indicating right-ward asymmetry (i.e., a greater right-ward than left-ward measure). ACC, anterior cingulate cortex; PCC, posterior cingulate cortex; OFC, orbitofrontal cortex; STG, superior temporal gyrus; IPC, inferior parietal cortex; fusiform, fusiform gyrus.

Figure 2 provides a schematic analysis of average brain asymmetries across all children. Left-ward asymmetry in cortical volume was observed in the STG, rostral ACC, and fusiform. Conversely, right-ward volume asymmetry was found in the IPC, caudal ACC, PCC, and medial OFC (Figure 2A). In terms of cortical thickness, conspicuous left-ward lateralization was evident in most of the fronto-limbic regions, except for the STG and rostral ACC (Figure 2B). Regarding the volume of subcortical regions, the thalamus and hippocampus showed prominent left-ward asymmetry, while the amygdala revealed right-ward asymmetry (Figure 2C).

### 3.4 Age-related structural asymmetries of the fronto-limbic volume and thickness

**Figure 3:**
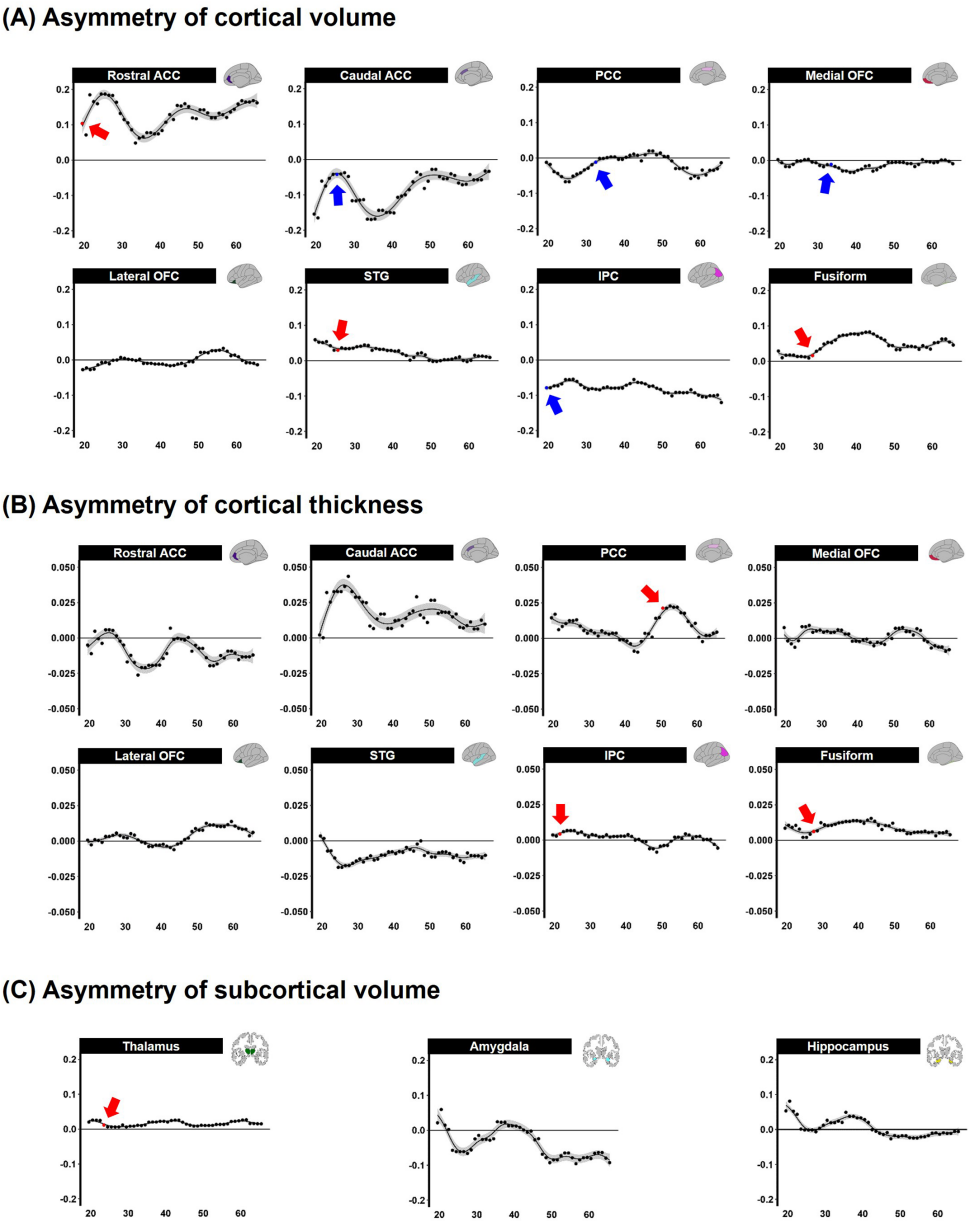
Sliding window analysis of normalized brain asymmetry. **(A)** Analysis of cortical gray matter volume asymmetry. **(B)** Analysis of cortical thickness asymmetry. **(C)** Analysis of subcortical gray matter volume asymmetry. All the points exhibit the asymmetry index calculated for each region within the sliding window analysis; positive score indicates left-ward asymmetry (Red point and arrow represent significant left asymmetry), while negative score indicates right-ward asymmetry (Blue point and arrow represent significant right asymmetry). ACC, anterior cingulate cortex; PCC, posterior cingulate cortex; OFC, orbitofrontal cortex; STG, superior temporal gyrus; IPC, inferior parietal cortex; fusiform, fusiform gyrus; FDR, false discovery rate; LSD, least significant difference.

The differences between the left and right hemispheres in age-related asymmetries are shown in Figure 3 and Table 2. Significant left-ward asymmetry of cortical volume was observed in the rostral ACC (*p* = 0.021), STG (*p* = 0.038), and fusiform (*p* = 0.020). In contrast, right-ward volume asymmetry was observed in the caudal ACC (*p* = 0.011), PCC (*p* = 0.041), medial OFC (*p* = 0.023), and IPC (*p* = 0.021) before the age of 5 years (Figure 3A). In cortical thickness, the PCC (*p* = 0.039), IPC (*p* = 0.047), and fusiform (*p* = 0.016) showed considerable left-ward lateralization before the age of 5 years (Figure 3B). Meanwhile, apparent asymmetry was not noted in the cortical thickness or volume of the lateral portions of the OFC. The volume of the subcortical regions in the thalamus was revealed (*p* = 0.027) (Figure 3C).

**Table 2:** Sliding window analysis of the fronto-limbic asymmetry.

ROI	Volume			Thickness		
	Asymmetry	Initial point (month)	FDR	Asymmetry	Initial point (month)	FDR
Rostral ACC	Left	19.5	**0**.**021**	NA	NA	NA
Caudal ACC	Right	25.5	**0**.**011**	NA	NA	NA
PCC	Right	32.5	**0**.**041**	Left	40.5	**0.039**
Medial OFC	Right	33.5	**0**.**023**	NA	NA	NA
Lateral OFC	NA	NA	NA	NA	NA	NA
STG	Left	25.5	**0**.**038**	NA	NA	NA
IPC	Right	19.5	**0**.**021**	Left	21.5	**0.047**
Fusiform	Left	28.5	**0**.**020**	Left	27.5	**0.016**
Thalamus	Left	23.5	**0**.**027**			
Hippocampus	NA	NA	NA			
Amygdala	NA	NA	NA			

Data are represented as significant initial points were defined by FDR corrected *p* < 0.05.

ROI, region of interest; FDR, false discovery rate; NA, not available; ACC, anterior cingulate cortex; PCC, posterior cingulate cortex; OFC, orbitofrontal cortex; STG, superior temporal gyrus; IPC, inferior parietal cortex; fusiform, fusiform gyrus.

Significant differences between the two hemispheres (*p* < 0.05) are highlighted in bold.

## 4 Discussion

This study revealed the normative trajectory and emergence of structural asymmetry in the fronto-limbic regions of preschool-aged children. Our study showed age-related variations in brain volume and thickness within distinct regions of the fronto-limbic. Cortical thinning of the bilateral medial OFC and right rostral ACC was significantly observed during preschool childhood. Additionally, our findings indicate a significant increase in bilateral amygdala and right hippocampus volume among preschool children, following an upward linear pattern. Regarding brain lateralization, remarkable asymmetry was detected in the volume of thalamus and the most of cortical regions excluding the lateral OFC. These results on typical brain development offer valuable insights for the timing of the fronto-limbic neurodevelopmental trajectory.

Our study demonstrated a progressive annual thinning of most fronto-limbic regions between the ages of 1 and 5. This observation aligns with previous research findings regarding the changes in cortical thickness during early childhood (10, 47). Cortical thinning is linked to intricate processes such as synaptic pruning and intracortical myelination (2, 48). Across early childhood, this phenomenon is considered a normal aspect of brain maturation (49). Of the fronto-limbic regions, our result revealed that cortical thickness of the bilateral medial OFC and right rostral ACC exhibited significant decreases with age. The cortical thickness of medial OFC stands out as the earliest region to reach its peak at the second year of age (12, 50), following a logarithmic decrease until 6 years of age (8). Interestingly, a study comparing developmental changes in children with ASD identified significant alterations in the cortical thickness of the medial OFC compared to healthy and typical children (51). These findings may suggest the importance and potential role of the medial OFC trajectory in delineating early growth in healthy and typical children, and emphasize the medial OFC as an indicator of neurodevelopmental disorders and normal early development. Additionally, STG in cortical thickness has been showed notable positive relationships with age in preschool children. The reason for these trends is unclear, but it could indicate a simultaneous logarithmic increase in cortical maturation and adjacent WM myelination from 1 to 6 years of age (8).

With the gray matter volume, there was significant association between age and the relative volume of the bilateral amygdala until 5 years of age, with steep positive association with age. A previous study involving 1–6-year-old children showed evidence of a significantly accelerating trend in amygdala volume at 4–5 years (12). Furthermore, another study focusing on individuals aged 8–30 demonstrated a consistent linear increase in amygdala volume with age (52). Considering that the most rapid and dramatic changes in subcortical volume occur during childhood (4), this study suggests that the observed increase in volume, reflecting amygdala maturation, may mark a pivotal point in functional significance from a typical preschool-age-related morphometric pattern.

Other than increased volume and thinned thickness, structural lateralization is a multifaceted phenomenon that facilitates effective information processing (25). The early stages of brain lateralization are closely tied to the subsequent maturation of specific cognitive and socio-emotional functions (25). Although various studies have investigated the asymmetric changes in the cerebral cortex across the lifespan (10, 53, 54), they have not identified the specific time points at which age-related changes in the brain thickness and volume of the fronto-limbic regions. We explored the asymmetry in volume and thickness within the fronto-limbic regions across ages. This study revealed that most fronto-limbic regions display a noticeable emergence of cortical volume asymmetry before the age of 5 years. This observation suggests that establishing lateralization in these regions may constitute a crucial structural and functional development stage. Regarding cortical volume, left-ward prominence was observed in the STG at the age of 2–3 years. During the early fetal development, specifically between 20 and 28 gestational weeks, right-ward volume asymmetry in the STG was evident in the specific posterior regions near the lateral temporal gyrus (55). However, the STG typically exhibits left-ward hemispheric volume asymmetry in both children and adults (54, 56). The results of this study suggest an asymmetric shift in the STG around 2–3 years of age; this may suggest a period of important change in cortical volume lateralization, shifting the right hemisphere to the left hemisphere. Moreover, the significant left-ward lateralization of the rostral ACC corresponds with the results of previous studies examining childhood (10) and adolescence (57), suggesting that structural and functional development can occur early after birth.

The majority of the fronto-limbic regions demonstrate asymmetric development in cortical volume before the age of 5, whereas significant asymmetry in cortical thickness is observed exclusively in the PCC, IPC and fusiform. The PCC is located in the medial portion of the inferior parietal lobe and is connected to the parietal lobe (58), where it receives information from it (59). The significant lateralization of the IPC and PCC is consistent with the functional development of the human brain, in which the sensory-motor systems are located posteriorly and mature before executive functions (5). Interestingly, the cingulate cortex, which is highly cytoarchitectonically and anatomically heterogeneous area (60, 61), exhibited different asymmetries in the same internal structures (62). Previous study found that brain metabolite ratios and types influenced gray matter development in adolescents (63). These are believed to indicate that the manifestation of divergent metabolite patterns influences gray matter development, leading to diverse asymmetries within specific internal structures (ie. caudal, rostral, and posterior). Notably, they revealed that cognitive variations, attributed to differences in both the types and levels of metabolites, became apparent in the PCC (63). On the other hand, the OFC did not show significant asymmetries in both cortical volume and thickness during childhood. These findings suggest that the frontal lobe undergoes a more prolonged maturation process involving both functional and structural reorganizations than the executive functions; moreover, functional specialization of the frontal lobe, marked by the emergence of cortical asymmetry, may signify a significant developmental milestone after preschool-aged children.

For subcortical volume asymmetry, our study demonstrated that the thalamus had significant left-ward asymmetry in preschool-aged children (age: 2 years), which is remarkably consistent in the left hemisphere from birth to adulthood (25, 64). Meanwhile, a study between childhood and adolescents with ASD identified atypical right-ward asymmetry in the thalamus as an important region underlying the anatomical basis of social deficits (65). These inconsistent findings may be related to alterations in the subcortical-cortical relays, potentially leading to changes in functional connectivity and thereby impacting social interactions (24). Although the generalizability of thalamic asymmetry has not been fully explored, this study highlights the importance of early stages in the structural and functional development of the thalamus in brain lateralization for developmental milestones.

In conclusion, we quantitatively analyzed trajectories of the cortical thickness, cortical volume and subcortical volume. Furthermore, we identified the emergence point of brain lateralization in the fronto-limbic regions during preschool-aged children. These findings suggest that the fronto-limbic structures of the preschool-aged period may be potentially associated with the cognitive and socio-emotional development in subsequent stage. Given that the fronto-limbic regions are closely associated with various neurodevelopmental disorders, identifying its trajectories and asymmetries provides critical insights regarding clinical development for preschool-aged children.

## 5 Limitation

This study has several limitations that should be addressed in future research. First, this study had a small sample size and analyzed a cross-sectional scan along with non-uniform sample sizes across different age groups, which may restrict the interpretation of the results. Second, a potential concern for our study is the use of FreeSurfer standard templates in preschool-aged children, which may result in inaccuracies in segmentation and parcellation. We should interpret our FreeSurfer analysis results in preschool-aged children with caution and consider using pediatric-specific tools or templates in future studies. Third, cognitive development is more effectively explained by systematic changes among various core regions than by the maturation of individual core structures. Hence, further study would be needed for a network-based approach, elucidating the maturational changes in structural connectivity by exploring the maturation of different core regions. Finally, analyzing cortical volume, thickness and subcortical volume as indicators of brain structure may not be sufficiently comprehensive in capturing the complexity of maturation that occurs in early childhood. Therefore, neuroimaging studies including various aspects of the brain metrics such as surface area, curvature and gyrification would be more valuable to achieve a comprehensive understanding the mechanisms of brain development.

## Data Availability

The original contributions presented in the study are included in the article/Supplementary Material, further inquiries can be directed to the corresponding authors.
