# Supplementary material for: Structural development and brain asymmetry in the fronto-limbic regions in preschool-aged children
Source: Front Pediatr. 2024 Oct 1;12:1362409. doi: 10.3389/fped.2024.1362409 (PMC11473423; doi:10.3389/fped.2024.1362409)
Supplement: Supplementary file 1 [file Datasheet1.pdf]

Supplementary Material

Development of volume and thickness in the structural fronto-limbic regions in preschool-aged children

Gang Yi Lee[1,†], Young-Ah Youn[2,†], Yong Hun Jang[1], Hyuna Kim[1], Joo Young Lee[1], Young Jun Lee[3], Minyoung Jung[4,‡,*], Hyun Ju Lee[5,6,7,‡,*]

†These authors contributed equally to this work.

‡ These authors contributed equally to this work and share the corresponding authorship.

*** Correspondence:** Corresponding Authors: 1) Minyoung Jung, minyoung@kbri.re.kr 2) Hyun Ju Lee, blesslee77@hanmail.net

Supplementary Figures

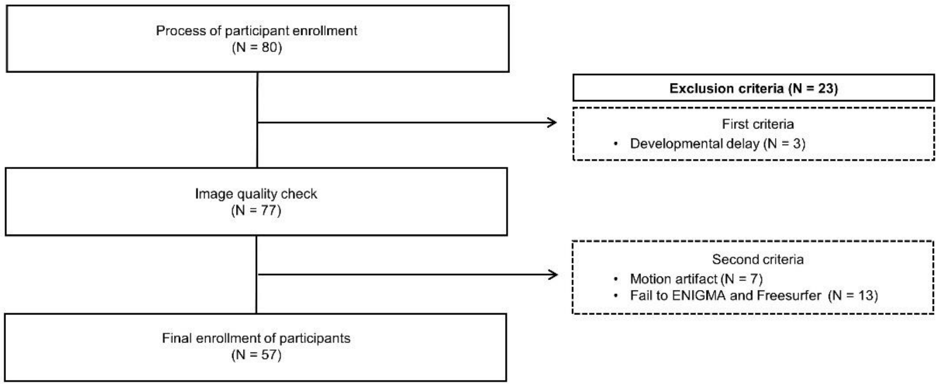

Supplementary Figure 1. Flowchart showing the participant selection process.

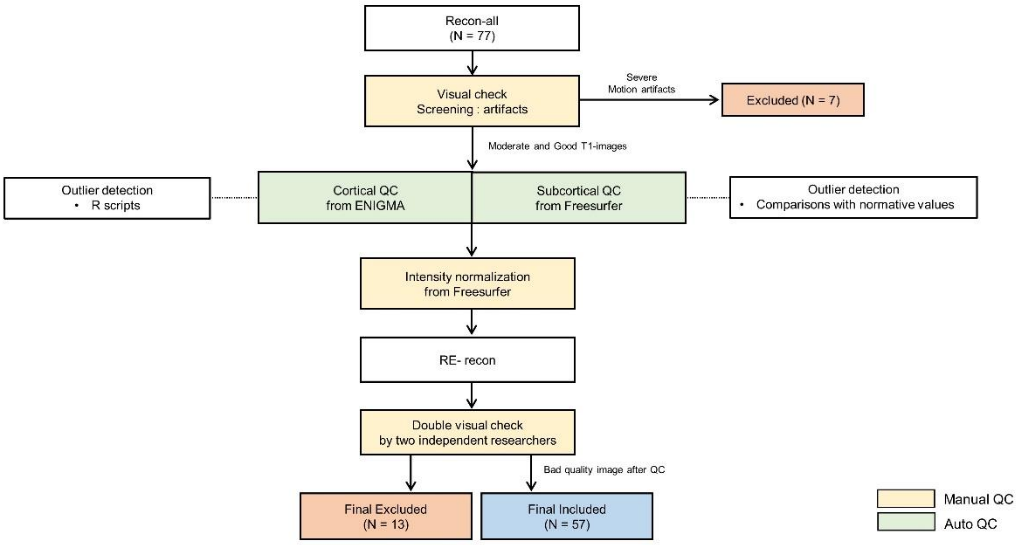

Supplementary Figure 2. Flowchart of the quality assessment of MR images.

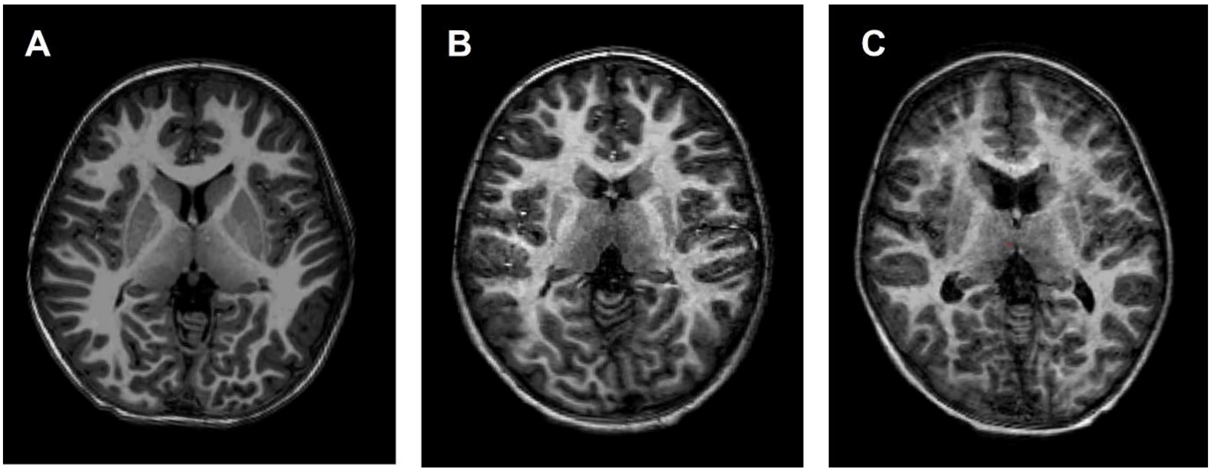

Supplementary Figure 3. Examples of T1-weighted images with each motion type in a dataset (A) good, (B) moderate, (C) bad.

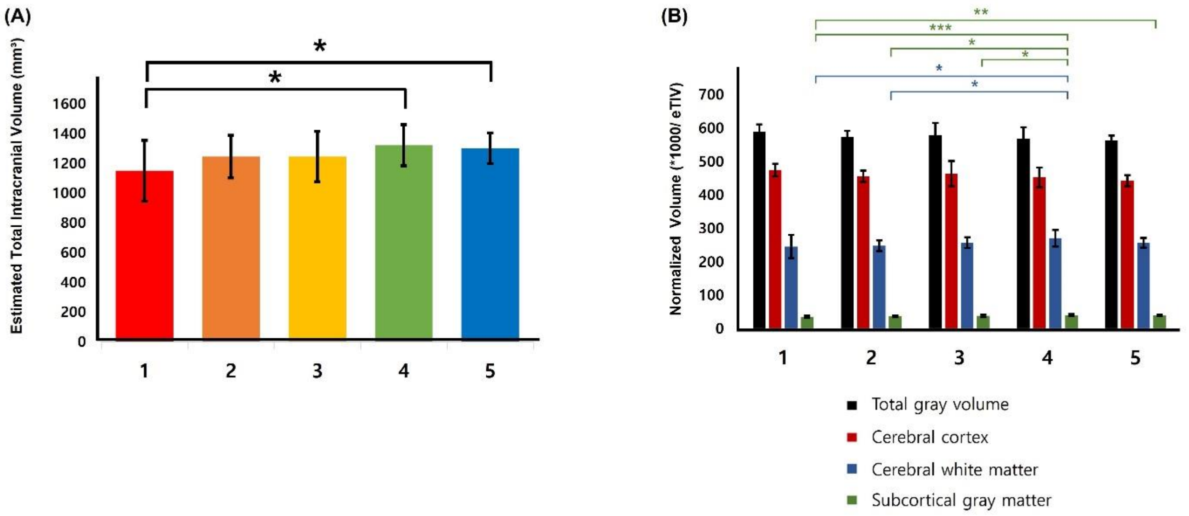

Supplementary Figure 4. Age-related variations in the volume of the whole brain and major types of brain tissues. **(A)** Total brain volume. **(B)** Normalized volume of the major types of brain tissues. The brain volume is normalized relative to the estimated total brain volume by dividing eTIV and multiplying by 1,000.

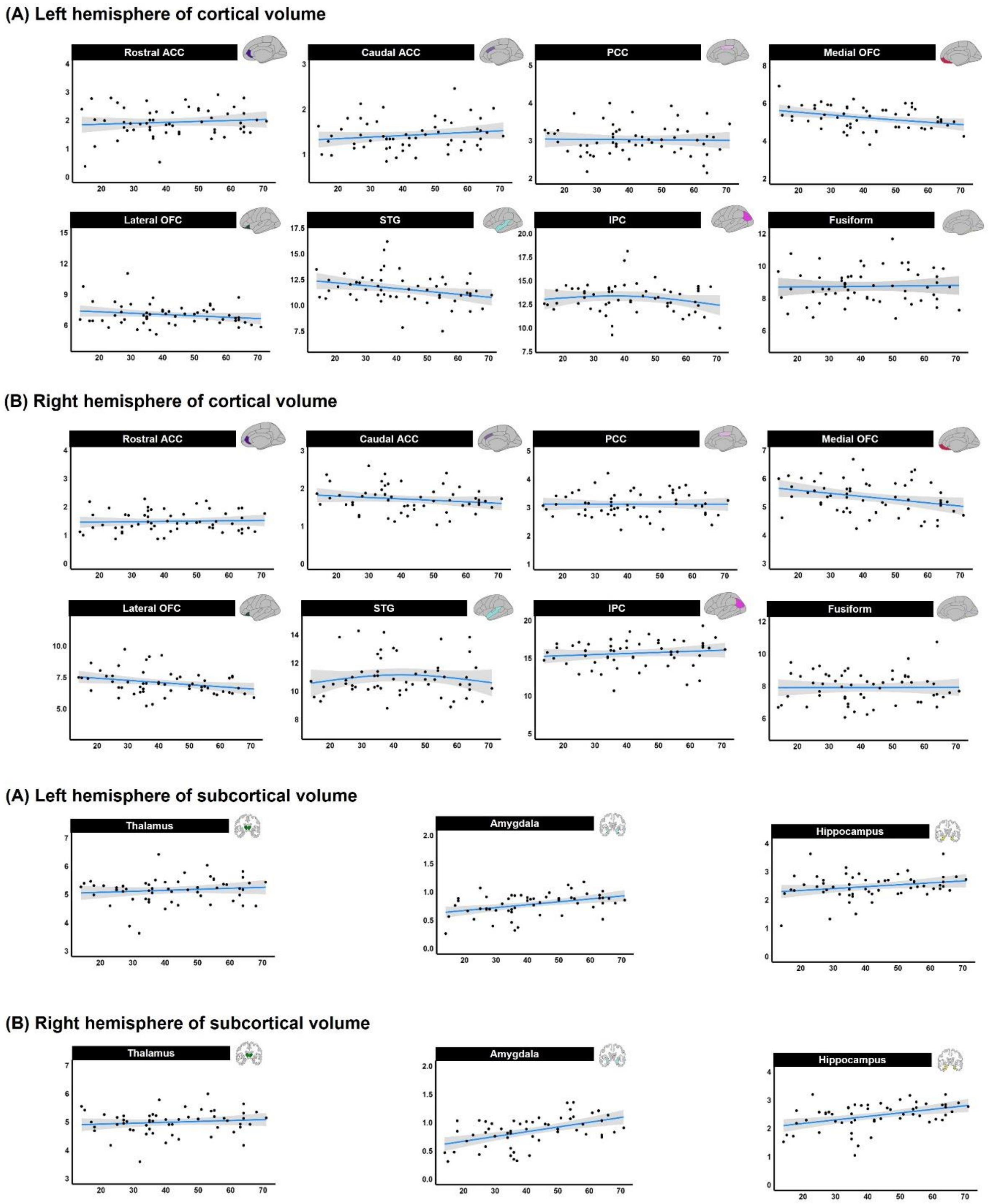

Supplementary Figure 5. Trajectory of region-specific cortical and subcortical volume development. **(A)** Trajectory of left hemisphere cortical volume. **(B)** Trajectory of right hemisphere cortical volume. **(C)** Trajectory of left hemisphere subcortical volume. **(D)** Trajectory of right hemisphere subcortical volume. Points correspond to subject specific volume value and the blue line corresponds to the trend line of best volume fit from BIC analysis. Abbreviations: ACC, anterior cingulate cortex; PCC, posterior cingulate cortex; OFC, orbitofrontal cortex; STG, superior temporal gyrus; IPC, inferior parietal cortex;

fusiform, fusiform gyrus.

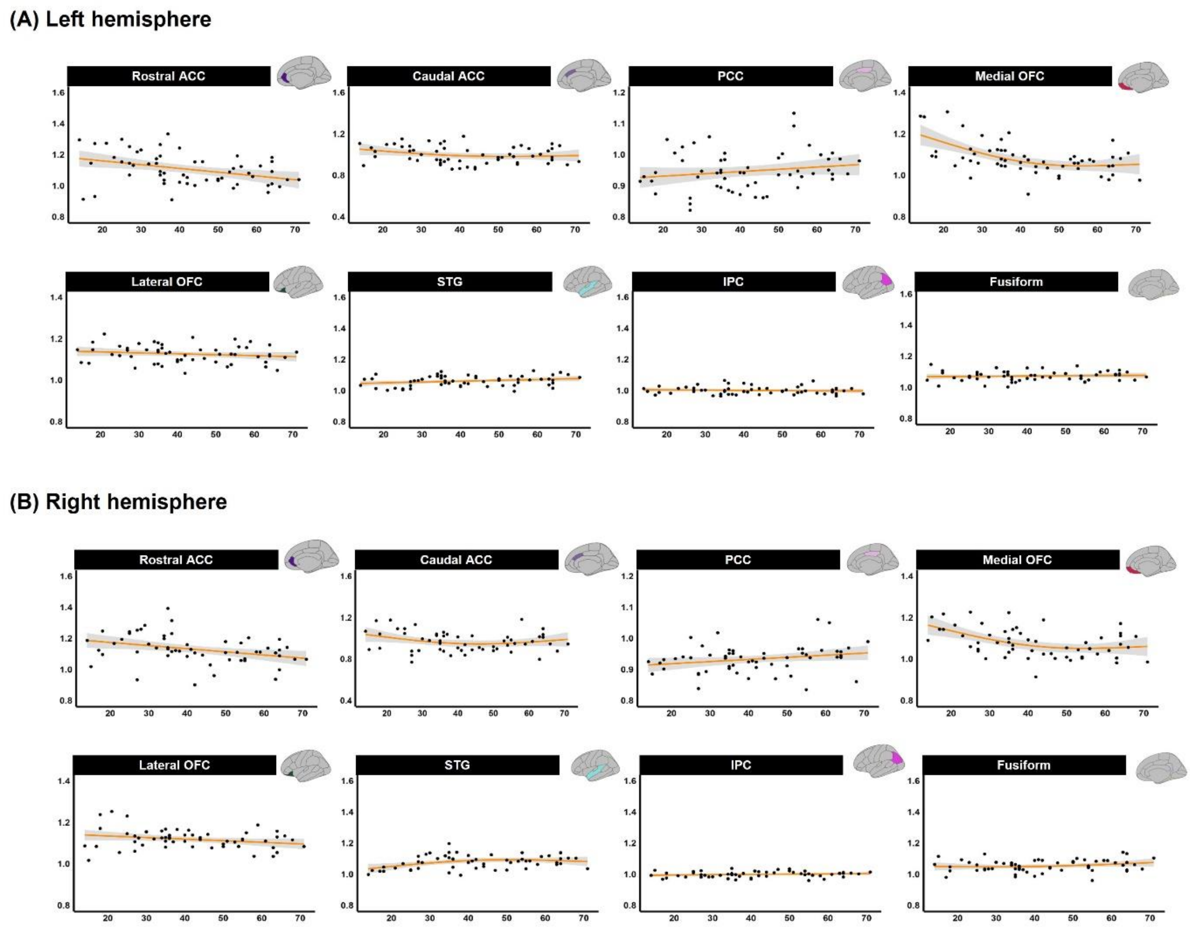

Supplementary Figure 6. Trajectory of region-specific cortical thickness development. **(A)** Trajectory of left hemisphere cortical thickness. **(B)** Trajectory of right hemisphere cortical thickness. Points correspond to subject specific volume value and the orange line corresponds to the trend line of best volume fit from BIC analysis. Abbreviations: ACC, anterior cingulate cortex; PCC, posterior cingulate cortex; OFC, orbitofrontal cortex; STG, superior temporal gyrus; IPC, inferior parietal cortex; fusiform, fusiform gyrus.

Supplementary Tables

Supplementary Table 1. Age-related absolute volume of the whole brain and major types of brain tissues.

	**Age 1 (n=7)**	**Age 2 (n=14)**	**Age 3 (n=15)**	**Age 4 (n=11)**	**Age 5 (n=10)**
**Absolute value**					
Total gray volume	676891.3	712043.3	721865.3	749864.8	732541.1
Cerebral cortex	569426.8	542475.4	577409	597141.4	599231.5
Cerebral white matter	288436.3	310239.5	322914	358183	335173.5
Subcortical gray matter	41863.57	46065.73	47928.71	53475.27	51921
Estimated total intracranial volume	1153127	1238812	1255122	1323209	1302482

Supplementary Table 2. GAM modeling statistics for normalized volume.

ROI	Left hemisphere				Right hemisphere			
	edf	F-value	*p*-value	FDR	edf	F-value	*p*-value	FDR
Rostral ACC	1.00	0.487	0.488	0.596	1.00	0.086	0.770	0.941
Caudal ACC	1.00	1.616	0.209	0.383	1.00	1.621	0.208	0.458
PCC	1.00	0.031	0.862	0.869	1.00	0.003	0.953	0.983
Medial OFC	1.00	7.179	0.010	0.055	1.00	5.644	0.021	0.077
Lateral OFC	1.00	2.042	0.159	0.350	1.00	4.72	0.034	0.094
STG	1.00	5.208	0.026	0.095	1.544	0.541	0.580	0.796
IPC	1.62	1.433	0.334	0.491	1.00	1.04	0.312	0.490
Fusiform	1.00	0.027	0.869	0.869	1.00	0	0.983	0.983
Thalamus	1.00	0.864	0.357	0.491	1.00	1.151	0.288	0.490
Hippocampus	1.00	3.096	0.084	0.231	1.00	11.65	0.001	**0.006****
Amygdala	1.00	10.74	0.002	**0.022***	1.00	19.04	<0.001	**0.001****

Effective degree of freedom (edf) and F-values in the GAM modeling of age x cortical thickness adjusted for sex. Significance is denoted with * and highlighted in bold. Abbreviations: edf, effective

degree of freedom; ACC, anterior cingulate cortex; PCC, posterior cingulate cortex; OFC, orbitofrontal cortex; STG, superior temporal gyrus; IPC, inferior parietal cortex; fusiform, fusiform gyrus.

Supplementary Table 3. GAM modeling statistics for normalized thickness**.**

ROI	Left hemisphere				Right hemisphere			
	edf	F-value	*p*-value	FDR	edf	F-value	*p*-value	FDR
Rostral ACC	1.00	8.369	0.005	0.020*	1.00	6.906	0.011	**0.032***
Caudal ACC	1.626	1.476	0.164	0.2832	1.769	1.81	0.137	0.1826
PCC	1.00	1.875	0.177	0.2832	1.00	3.303	0.075	0.12
Medial OFC	1.839	11.88	<0.001	**<0.001*** **	1.792	6.542	0.002	**0.016***
Lateral OFC	1.00	1.46	0.232	0.3093	1.00	3.605	0.063	0.12
STG	1.00	3.631	0.062	0.165	2.651	4.56	0.012	**0.032***
IPC	1.00	0.332	0.567	0.603	1.00	1.595	0.212	0.2422
Fusiform	1.00	0.273	0.603	0.603	1.459	1.994	0.250	0.25

Effective degree of freedom (edf), F-values in the GAM modeling of age x cortical thickness. Significance is denoted with * and highlighted in bold. Abbreviations: edf, effective degree of freedom; ACC, anterior cingulate cortex; PCC, posterior cingulate cortex; OFC, orbitofrontal cortex; STG, superior temporal gyrus; IPC, inferior parietal cortex; fusiform, fusiform gyrus.
